# Enhanced oxidation resistance of active nanostructures via dynamic size effect

**DOI:** 10.1038/ncomms14459

**Published:** 2017-02-22

**Authors:** Yun Liu, Fan Yang, Yi Zhang, Jianping Xiao, Liang Yu, Qingfei Liu, Yanxiao Ning, Zhiwen Zhou, Hao Chen, Wugen Huang, Ping Liu, Xinhe Bao

**Affiliations:** 1 State Key Laboratory of Catalysis, CAS Center for Excellence in Nanoscience, Collaborative Innovation Center of Chemistry for Energy Materials, Dalian Institute of Chemical Physics, Chinese Academy of Sciences, Zhongshan Road 457, Dalian 116023, China; 2 University of Chinese Academy of Sciences, Beijing 100049, China; 3 Chemistry Department, Brookhaven National Laboratory, Upton, New York 11973, USA

## Abstract

A major challenge limiting the practical applications of nanomaterials is that the activities of nanostructures (NSs) increase with reduced size, often sacrificing their stability in the chemical environment. Under oxidative conditions, NSs with smaller sizes and higher defect densities are commonly expected to oxidize more easily, since high-concentration defects can facilitate oxidation by enhancing the reactivity with O_2_ and providing a fast channel for oxygen incorporation. Here, using FeO NSs as an example, we show to the contrary, that reducing the size of active NSs can drastically increase their oxidation resistance. A maximum oxidation resistance is found for FeO NSs with dimensions below 3.2 nm. Rather than being determined by the structure or electronic properties of active sites, the enhanced oxidation resistance originates from the size-dependent structural dynamics of FeO NSs in O_2_. We find this dynamic size effect to govern the chemical properties of active NSs.

## Abstract (web summary)

Smaller nanostructures often have higher activities, but at the expense of losing chemical stability. Here, the authors find that reducing the size of FeO nanostructures to below 3 nm enhances their resistance to oxidation, and attribute this unexpected observation to a size-dependent, structural dynamics effect.

Nanostructures (NSs) of a few nanometres in size often exhibit prominent size-dependent properties^1^,^2^,^3^,^4^,^5^. With reducing size, surface defects become more prominent, and the electronic structure of NSs can fluctuate due to electron confinement^2^,^3^,^5^,^6^. Thus, the origin of size effects in chemical reactions has usually been attributed to the size-dependent geometric structures or electronic properties, which have been measured statically and in the absence of reactant molecules^2^,^3^,^6^. Much less is known regarding the dynamic interaction between NSs and reactant molecules, despite its essential role in the reaction^7^. Among the large volume of studies on the oxidation of NSs, detailed studies on the oxidation mechanism, especially on developing a fundamental understanding of oxidation kinetics, have been very limited^8^,^9^. The oxidation of NSs has often been described by the Cabrera–Mott model^10^,^11^, which suggests that the growth rate or the thickness of oxide shell is inversely dependent on the size of NS^12^. A recent atomic study on the oxidation of Fe NSs has shown, however, that the Cabrera–Mott theory^11^ might not be accurate to explain the oxidation of NSs and the formation of oxide shells could facilitate metal oxidation via the strain-mediated ionic transport^13^, surpassing the prediction by the Cabrera–Mott model.

Indeed, the studies of size effects on oxidation kinetics have been particularly difficult because of the structural variations among NSs introduced when producing different-sized NSs^9^. Several nanocrystalline materials were reported previously to exhibit improved oxidation resistance with respect to bulk materials and have been applied as anti-corrosion coatings on steels^14^,^15^. The underlying mechanism, however, is not well understood, owing to the lack of atomic understanding on their interaction with O_2_. Thus, there have been no general consensus on the oxidation resistance of oxide NSs as to the effect of NS size^9^. Particularly, the oxidation kinetics of NSs with diameters <5 nm have rarely been studied or reported. In catalysis, NSs with diameters below 3 nm often exhibit exceptional catalytic properties, whose nature has remained largely unknown^2^,^5^.

Fe and Fe oxide NSs have been widely explored as candidate materials in energy, environment and health technologies^13^,^16^,^17^. When pristine Fe is exposed to O_2_, FeO forms spontaneously^18^ and is the desired phase for applications in catalysis^19^,^20^, lithium batteries^21^, optics^22^ and magnetic devices^23^,^24^,^25^. FeO NSs supported on Pt have also been shown as a remarkable catalyst for the preferential oxidation of CO at low temperatures^19^. However, FeO is susceptible to further oxidation in an oxidative environment, which has been a major obstacle in the development of FeO-based functional materials^16^,^26^.

In this work, combining *in situ* scanning tunnelling microscopy (STM) and density functional theory (DFT) methods, we report a detailed study on the size-dependent oxidation of FeO NSs. By resolving the interaction between different-sized NSs and O_2_ at the atomic scale, we demonstrate a dynamic size effect, which is dominant for NSs with dimensions below 3 nm and could occur on NSs with the same atomic structure or electronic properties.

## Results

### Size-dependent oxidation of supported FeO NSs

**Figure 1:**
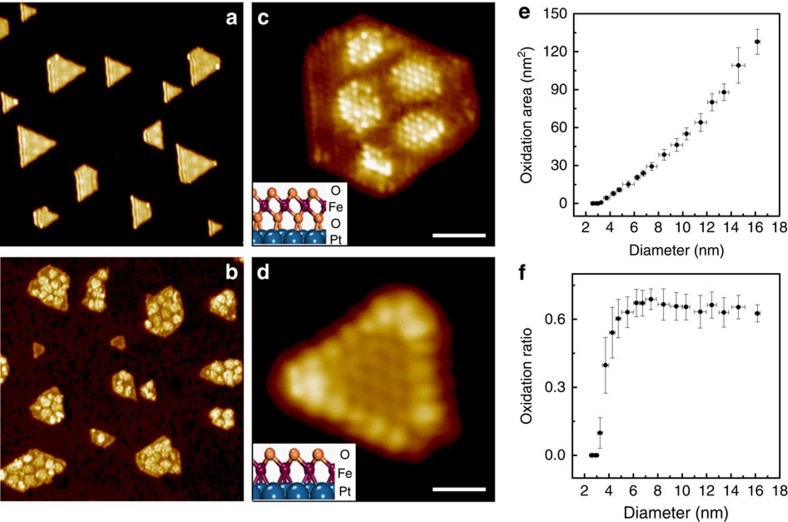
The size-dependent oxidation kinetics of FeO nanostructures(NSs) on Pt(111). (**a**,**b**) STM images (50 nm × 50 nm) of the typical surface of as-prepared FeO NSs on Pt(111) (**a**) and the FeO/Pt(111) surface after the annealing in 1 × 10^−5^ mbar O_2_ at 500 K for 10 min (**b**). Most FeO NSs were oxidized to form FeO_2_ domains, except for FeO NSs with an equivalent diameter *d*<3.2 nm. Here, the FeO NS is treated as a circle and the equivalent diameter is defined as 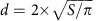, where *S* is the NS surface area. (**c**,**d**) Atomic STM images of an oxidized FeO NS (*d*=6.2 nm) with the formation of FeO_2_ domains and an FeO NS (*d=*2.1 nm) remaining the FeO phase, respectively. The structural models (side view) of FeO_2_ and FeO on Pt(111) are shown in the insets. STM images were taken at 230 K and scanning parameters (sample bias: *V*_s_; tunneling current: *I*_t_) are (**c**) *V*_s_=+104 mV, *I*_t_=3.5 nA; (**d**) *V*_s_=+53 mV, *I*_t_=2.1 nA. Scale bars are 2 nm in **c** and 1 nm in **d**. The area of FeO_2_ domains (*S*_FeO2_) on each NS, as well as the NS surface area (*S*), are measured over 247 FeO islands on the same surface. (**e**,**f**) plot the oxidation area (*S*_FeO2_) and the oxidation ratio (*S*_FeO2_/*S*) of individual FeO NSs as a function of *d*, where the size range of FeO NSs and the s.d. of *S*_FeO2_ and *S*_FeO2_/*S* are represented by error bars.

FeO NSs were prepared on Pt(111) and display typically the shapes of triangles or hexagons (Fig. 1a). The structure of FeO islands on Pt(111) has been well characterized as a polar FeO(111) bilayer, with the Fe layer in contact with Pt and the O layer exposed at the topmost plane^27^,^28^,^29^,^30^. Due to lattice mismatch, Fe atoms occupy successively the fcc, hcp and top positions on Pt(111), forming moiré domains on FeO (ref. 31). When exposed to O_2_ at elevated temperatures, FeO NSs could be oxidized by oxygen penetration into the FeO/Pt interface to form FeO_2_ (Fig. 1b), displaying an O–Fe–O trilayer structure^26^. DFT calculations suggested that the FeO_2_ phase was thermodynamically more stable than FeO on Pt(111) (ref. 32). The structural difference between FeO_2_ and FeO could be easily distinguished in STM from their apparent heights, moiré patterns and atomic structures (Supplementary Fig. 1).

Interestingly, the oxidation kinetics of FeO NSs at 500 K or below were found to be strongly size-dependent (Fig. 1b). While most FeO islands were oxidized to form FeO_2_ (Fig. 1c), small FeO islands remained the FeO phase (Fig. 1d). Figure 1e shows that the area of FeO_2_ domains on an individual island increases with the increasing island size (*d*). No FeO_2_ domains were observed on FeO islands with *d*<3.2 nm (Supplementary Fig. 2). *In situ* STM images (Supplementary Fig. 2) show that FeO_2_ domains were developed by oxygen penetration from the step edges of FeO NSs, as generally proposed in the oxidation of NSs^9^. Assuming a uniform diffusion rate for oxygen insertion, the formation rate of FeO_2_ domains should be proportional with the edge perimeters of FeO NSs and smaller FeO NSs should display a higher oxidation ratio (Supplementary Fig. 2c). In contrast, we found the oxidation ratio of FeO NSs went down drastically for islands with *d*<3.2 nm (Fig. 1f).

### Size-dependent structural dynamics of supported FeO NSs in O_2_

**Figure 2:**
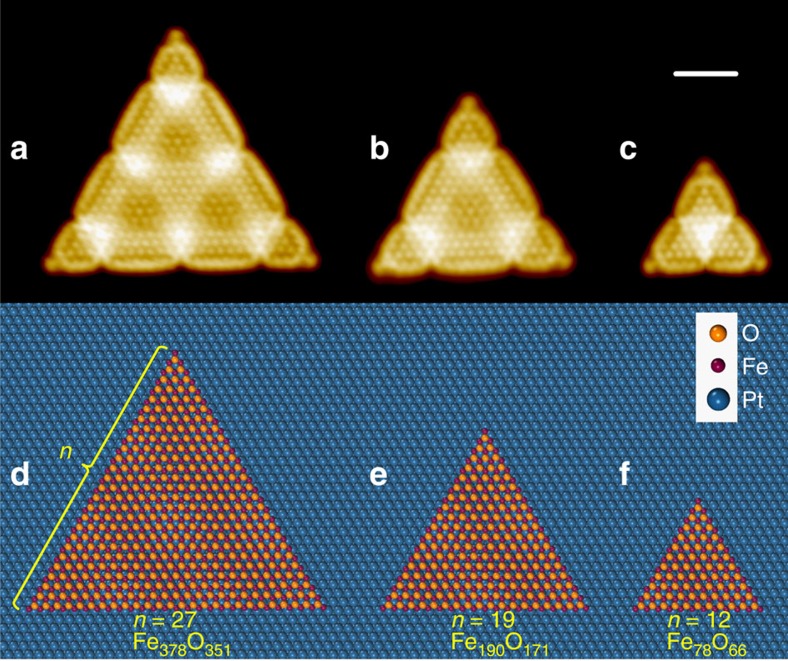
STM images and the corresponding structural models of equilateral-triangle-shape FeO NSs. In the structural models (**d**–**f**), the number of Fe atoms along each edge of the FeO triangle is denoted by *n*. The atomic compositions and structures of three FeO NSs are obtained from STM images (**a**–**c**). STM images were taken at 5 K and scanning parameters are (**a**) *V*_s_=+6 mV, *I*_t_=5.5 nA; (**b**) *V*_s_=+7 mV, *I*_t_=5.9 nA; and (**c**) *V*_s_=+7 mV, *I*_t_=4.5 nA. Scale bar, 2 nm.

By examining the structure of FeO NSs and their interaction with O_2_, we found the enhanced oxidation resistance of FeO NSs with *d*<3.2 nm was caused by a dynamic size effect described below. As-prepared FeO NSs typically exhibit two types of step structures, exposing two-coordinated Fe or O atoms (Supplementary Figs 3 and 4). The steps terminated by Fe atoms, also known as coordinatively unsaturated ferrous (CUF) sites, are active sites for O_2_ dissociation^19^,^33^, whereas the steps terminated by coordinatively unsaturated oxygen (CUO) atoms and the surface plane of FeO were found inert to O_2_ (ref. 16). For simplification, the two types of steps were termed as the CUF step or the CUO step, whose atomic structures could be viewed directly in STM images (Supplementary Fig. 4). A detailed structural analysis showed that the structure of the CUF step is independent on the island size. As manifested in Fig. 2, three triangular FeO islands expose exclusively CUF steps, which display the same structure and lattice constant.

**Figure 3:**
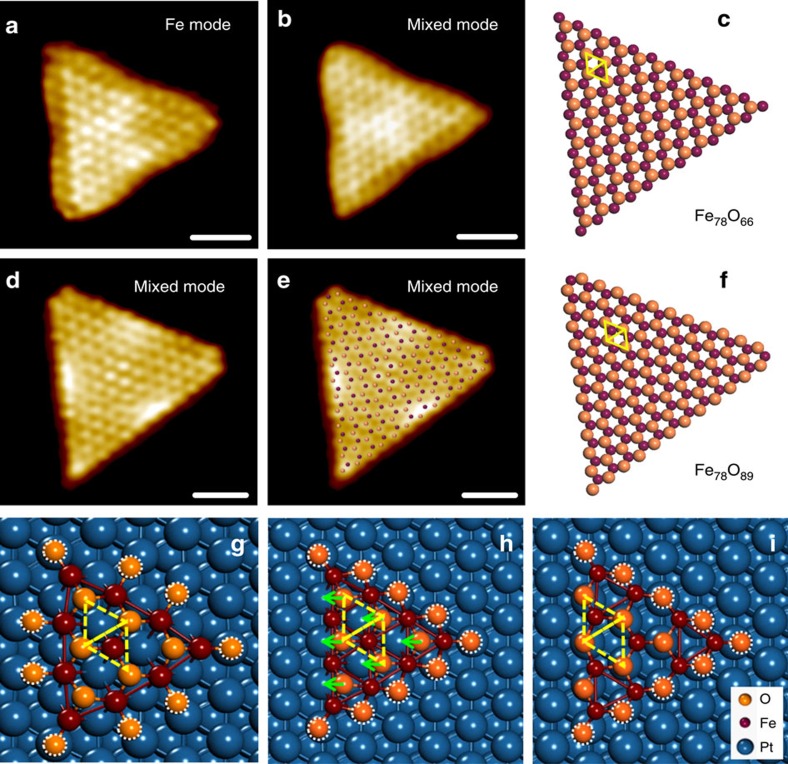
The structural dynamics of FeO NSs in O_2_. (**a**–**f**) *In situ* STM images and the corresponding structural models of an Fe_78_O_66_ NS before (**a**–**c**) and after (**d**–**f**) the exposure of 1 × 10^−9^ mbar O_2_ at 270 K. Element-specific STM images were used to identify the number of Fe and O atoms, as well as their relative positions. (**a**,**b**) are the Fe-mode and mix-mode STM images of the Fe_78_O_66_ NS at 270 K, which underwent a complete reconstruction in O_2_ and turned into an Fe_78_O_89_ island. (**d**) Mix-mode STM image of the Fe_78_O_89_ island, which is overlayed with the structural model in **e**. In the mix-mode images, O atoms are resolved as bright protrusions and the hollow sites of the Fe lattice, which are not filled by O atoms, are resolved as dark depressions. The positions of dark depressions with respect to O atoms have changed after O_2_ exposure, which is illustrated in the structural models in **c**,**f**, with colour representations of: Fe—purple, O—orange and Pt—blue. STM images were taken at 270 K and scanning parameters are (**a**) *V*_s_=+16 mV, *I*_t_=4.3 nA; (**b**) *V*_s_=+34 mV, *I*_t_=1.9 nA; (**d**) and (**e**) *V*_s_=+80 mV, *I*_t_=1.8 nA. Scale bars are 1 nm for all STM images. (**g**–**i**) Calculated adsorption configurations of O atoms at the edges of an Fe_10_O_15_ cluster on Pt(111). Among the three configurations, oxygen adatoms bind most weakly in **g** and most strongly in **i**. Compared with **g**, the configurations in **h**,**i** have energy gains of −2.10 and −2.79 eV, respectively. To reach **i**, oxygen atoms in **h** need be shifted, with the moving directions marked by green arrows.

Despite the same shape and structure, these triangular FeO islands exhibited a drastically different structural dynamics when exposed to O_2_ at 270 K. Figure 3 shows O_2_ dissociation at CUF sites of the Fe_78_O_66_ NS led to the spontaneous and complete reconstruction of NS, which was also the case for smaller FeO NSs (Supplementary Fig. 5). Element-specific STM images (Supplementary Note 1) have allowed us to reveal not only the number of Fe and O atoms, but also their relative positions. Upon O_2_ exposure, 23 O atoms were added to the edges of Fe_78_O_66_ NS, accompanying a collective shift of all oxygen atoms to the adjacent 3-fold hollow sites of the Fe layer (Supplementary Note 2, Supplementary Figs 6 and 7). Consequently, all CUF sites at the edges could be saturated by two-coordinated oxygen atoms, forming the CUO steps rather than by dangling oxygen atoms as before reconstruction. The line profile of the CUO step appears inverted to that of the CUF step (Supplementary Fig. 7). DFT calculations show that, before reconstruction, dangling oxygen atoms bind weakly at the step edge, with the adsorption energy of 0.64 eV (Fig. 3g). The binding energy of O adatoms is increased to 1.26 eV by bonding with two neighbouring Fe atoms (Fig. 3h), though such a configuration is not stable and prefers to reconstruct. The reconstruction shifts surface O atoms to the adjacent three-fold hollow sites of Fe lattice and results in the strong binding of edge O atoms with the adsorption energy of 2.14 eV (Fig. 3i). Thus, all O adatoms were stabilized at the step edges by the spontaneous reconstruction of FeO NSs.

In contrast, a partial reconstruction was observed for larger NSs, such as Fe_210_O_190_ and Fe_378_O_351_. Supplementary Figure 8 shows only a portion of surface oxygen atoms was shifted to the adjacent 3-fold hollow sites of Fe lattice, resulting in a reconstructed oxygen domain and oxygen dislocation lines at the boundary between reconstructed/unreconstructed domains. At the dislocation, Fe atoms were over-saturated with 4-fold oxygen coordination and appeared as protrusion lines running parallel to the steps (Supplementary Fig. 8)^34^. The reconstructed domain was evidenced by the formation of CUO steps, while the edges of unreconstructed domain were terminated by either dangling or dislocated oxygen atoms^19^,^34^.

The size-dependent structural dynamics of FeO NSs could be driven via different channels. DFT calculations on supported FeO clusters show that, while the energy released from oxygen adsorption increases with the size increasing from Fe_10_O_6_ to Fe_28_O_21_, the driving force (or thermodynamic preference) associated with the shift of oxygen atoms to achieve a complete reconstruction decreases more rapidly (Supplementary Fig. 9). Both energy release and the driving force will eventually level off with increasing size. These results suggest that a complete structural reconstruction likely occurs in small NSs, which is in agreement with STM study. For example, although the energy release from oxygen adsorption at Fe_10_O_6_/Pt(111) is the smallest among the FeO clusters being calculated, its reconstruction is thermodynamically most favourable. On the other hand, with size increasing, the increment in the energy released from oxygen adsorption cannot match the decrease in the driving force from reconstruction (Supplementary Fig. 9), and thus a complete reconstruction becomes less favourable. Indeed, STM study shows that the reconstruction of larger FeO NSs is only partial with the formation of surface dislocations (Supplementary Fig. 8).

**Figure 4:**
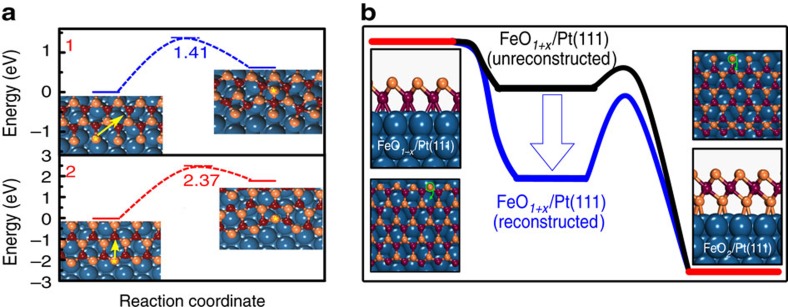
Dynamic size effect on the oxidation of FeO NSs. (**a**) Potential energy diagram depicting the mode of action for oxygen reconstruction. The diffusion pathways and barriers are displayed for oxygen penetration into the FeO–Pt(111) interface from the unreconstructed edge (1) and the reconstructed CUO edge (2). (**b**) A schematic illustration of the dynamic size effect in enhancing the oxidation resistance of active FeO NSs. The dynamic response of FeO NS enables FeO to reach an intermediate state, which has a lower total energy than that before the reconstruction and thus increases the barrier for further oxidation.

The dynamic response of FeO NSs could be observed at even 15 K, upon the dissociative adsorption of oxygen at the CUF sites (Supplementary Fig. 10). The fact that an adsorption of several oxygen atoms at the edge of FeO NSs could induce locally the reconstruction of FeO NSs at such a low temperature suggests that the reconstruction could be initiated without thermal activation. However, DFT calculations show that, to initiate the reconstruction, diffusion of isolated oxygen atoms to adjacent 3-fold hollow sites of Fe needs to overcome a barrier (*E*_d_) of ∼0.33 eV/O atom (Supplementary Fig. 11). Meanwhile, oxygen insertion into the FeO–Pt(111) interface, that is, the transition to FeO_2_, has an even higher energy barrier of 1.41 eV/O atom (Fig. 4a). Thus, the driving force for the reconstruction is unlikely to be limited to thermodynamics.

Note that we have not taken into account the partitioning of the energy released from oxygen adsorption, which should be in the form of hot electrons^35^ and local atomic displacements. The energy channelling model requires that these energies are effectively used for the reconstruction, rather than generating thermal losses^36^. It is believed that hot electrons typically decay on the timescale of picoseconds. Such a fast timescale could in principle inhibit the channelling to take place, or alternatively be the reason why the reconstruction of smaller FeO NSs is complete but not the larger ones. In any case, the concerted reconstruction of NSs is a complex process; their activation energies depend on the density of the CUF sites (that is, the number of adsorbed oxygen atoms) and the size of the NSs (Supplementary Fig. 11). As such, we cannot rule out the possibility of barrierless channels upon saturation adsorption of oxygen at the CUF sites. This size-dependent structural dynamics warrants further exploration both experimentally and theoretically using time-dependent approaches to gain in-depth understanding.

### The oxidation of FeO NSs determined by dynamic size effect

The dynamic size effect governs the oxidation kinetics of FeO NSs by tuning the stability of O atoms at the step edges. *In situ* STM images (Supplementary Fig. 2) showed that the development of FeO_2_ domains along step edges was indeed anisotropic and controlled by the step structures of FeO NSs. Once the oxidation has been initiated and oxygen entered the interface, we observed spontaneously the formation of FeO_2_ domains, as modulated by the interface. Meanwhile, previous studies have also suggested that the oxidation of Fe-hcp domain^26^ or Fe-fcc domain^37^ to form FeO_2_ is thermodynamically most favourable, which means the enhanced oxidation resistance of FeO NSs is not controlled by the different stability of FeO domains since FeO NSs with size below 5 nm consist of mostly Fe-hcp and Fe-fcc domains. In addition, DFT calculations suggested that the reconstruction to CUO-termination hindered significantly the diffusion of edge oxygen atoms to the FeO–Pt(111) interface (barrier of 2.37 eV/O atom, Fig. 4a) and consequently prevented the oxidation to form FeO_2_. In comparison, the diffusion barriers for oxygen at the unreconstructed edge (barrier of 1.41 eV/O atom, Fig. 4a) and surface oxygen dislocations (barrier of 0.50 eV/O atom, Supplementary Fig. 11) were much lower. Thus, FeO NSs with *d*<3.2 nm are likely passivated from oxidation by stabilizing all oxygen adatoms and forming CUO steps via the complete reconstruction in O_2_. In contrast, the partial reconstruction of larger FeO NS resulted in unreconstructed steps and the development of oxygen dislocation lines, both of which are vulnerable for oxygen penetration (Supplementary Fig. 11). Thermodynamically, the dynamic response of FeO NS enables FeO to reach an intermediate state, which has a lower total energy than that before the reconstruction and thus increases the barrier for further oxidation (Fig. 4b). The long-term stability of FeO NS in O_2_ is thus not dependent on the structure of active sites, but rather determined by the dynamic size effect.

### The generality and implications of dynamic size effect

The enhanced oxidation resistance was also found for CoO NSs with *d*<3 nm supported on Pt(111) and Au(111), whereas larger CoO NSs are susceptible for further oxidation (Supplementary Fig. 12). Thus, the enhanced oxidation resistance of smaller active NSs is not just a unique feature of the FeO/Pt(111) system, but could rather be observed in other supported NSs. We expect that the reconstruction mechanism discussed above could be transferred to supported active NSs with similar structural configuration. Indeed, a number of rocksalt-type oxides, such as FeO, CoO, NiO, MnO, VO and EuO, have been shown to exhibit similar structural configurations, when they were supported on different metal substrates, such as Pd, Rh, Pt, Au and Ag^38^,^39^,^40^. These supported oxide NSs are promising for a number of applications in catalysis, magnetic storage and material sciences^21^,^24^,^39^,^40^,^41^.

Oxides are usually considered as a rigid surface during the reaction at low temperatures^42^. We show that oxide NS can exhibit a rapid structural change at the elementary step. The triangular FeO NSs investigated above exhibit not only the same shape and structure, but also similar electronic properties and electrostatic potential (Supplementary Figs 9 and 13), which usually indicate their similar behaviour in oxidation. Instead, the dynamic size effect observed here manifests its dominant influence in the nanoscale chemistry. Although we have used a model system to illustrate the dynamic size effect, this effect should be somewhat general for active NS in exothermic reactions. Our results demonstrate how NS prevents the insertion of oxygen into the oxide–metal interface, which might be key to develop passivating coatings for metals.

In summary, we demonstrate a dynamic size effect that governs the oxidation of active FeO NSs. Even with the same structure, FeO NSs exhibited a size-dependent structural dynamics when reacting with O_2_. The structural dynamics is driven by the exothermic reaction between O_2_ and CUF sites. Since the density of active CUF sites decrease with the increasing size of NS, the reaction energies are only sufficient for the complete reconstruction of FeO NSs with *d*<3.2 nm, but fall short for larger ones. As a result, FeO NSs with *d*<3.2 nm could stabilize oxygen at the steps and passivate themselves from oxidation, whereas larger FeO NSs suffer deep oxidation due to the partial reconstruction and the development of dislocation lines. The same enhanced oxidation resistance was also observed for CoO NSs with *d*<3 nm, indicating the dynamic size effect could be general in the oxidation of supported active NSs, at least for those with similar structural configurations. Therefore, our findings provide not only a general understanding for the enhanced oxidation resistance in nanomaterials, but also new routes for stabilizing active nanocrystals and developing oxidation-resistant coatings.

## Methods

### Experimental details

The experiments were carried out in a combined ultrahigh vacuum (UHV) system equipped with Createc low-temperature scanning tunnelling microscope (LT-STM), XPS, UPS and the cleaning facilities. The STM and preparation chambers have a base pressures of 4 × 10^−11^ mbar and 6 × 10^−11^ mbar, respectively. The Pt(111) single crystal (Matek) was cleaned by cycles of Ar ion sputtering (1.5 keV, 10 μA) and annealing at 1,200 K. Nano-sized FeO islands were deposited onto Pt(111) by vapour deposition of Fe atoms in an O_2_ atmosphere (P(O_2_)=1 × 10^−7^ Torr) with the temperature of Pt(111) held at 300 K. The as-deposited surface was then annealed in UHV between 500–600 K, leading to the formation of well-ordered FeO nanocrystals. CoO islands were deposited on Pt(111) and Au(111) in P(O_2_)=1 × 10^−6^ Torr with the temperature of Pt(111) and Au(111) held at 300 and 400 K, respectively. The as-deposited surface was then annealed at 600 K (ref. 43).

The prepared sample was then transferred to the STM chamber where the surface could stay clean for days within the shields of the cryostat. Surface cleanness was verified by STM. STM images could be acquired at variable temperatures. To reach 5 K, the cryostat of the STM chamber was filled with liquid helium; to reach 78 K, the cryostat of the STM chamber was filled with liquid nitrogen. For STM measurements at temperatures higher than that of the cryostat, an on-stage Zenor diode was used to heat the STM stage to the desired temperature. For STM measurements above 150 K, the cryostat was filled with cold-bath liquids with melting points close to our desired temperature to maintain a pseudo-isothermal environment for stable STM measurements.

A second system equipped with near-ambient pressure (NAP) STM (SPECS, base pressure<2 × 10^−10^ mbar) was also used for *in situ* studies of oxidation. The preparation method of the FeO/Pt(111) sample was the same as described above. STM images were obtained at 300 K using a Pt-Ir tip in the NAP-STM chamber. STM images were processed with the SPIP programme from Image Metrology.

### Theoretical calculations

Computational analysis was performed based on DFT calculations, as implemented in Vienna ab initio simulation packages (VASP)^44^. The projector augmented wave scheme^45^ and Perdew–Burke–Ernzerhof^46^ functional was adopted for geometric optimizations. The calculated Hellmann–Feynman forces were specified to be smaller than 0.05 eV Å^−1^ in geometric optimization. The kinetic energy cutoff of 400 eV was chosen for the plane wave expansion. The vacuum space between two images was specified to be ∼10 Å. In addition, the Brillouin zones were sampled by gamma point as the unit cell is sufficiently huge. The on-site coulomb and exchange interactions for Fe atoms are specified to be 4.0 and 1.0 eV, respectively. The kinetic barrier of oxygen migration was calculated by constraint optimization via fixing the motion of oxygen in a specific direction.

### Data availability

The data that support the findings of this study are available from the corresponding authors upon reasonable request.

## Additional information

**How to cite this article:** Liu, Y. *et al*. Enhanced oxidation resistance of active nanostructures via dynamic size effect. *Nat. Commun.* **8**, 14459 doi: 10.1038/ncomms14459 (2017).

**Publisher's note:** Springer Nature remains neutral with regard to jurisdictional claims in published maps and institutional affiliations.

## Supplementary Material

Supplementary Information Supplementary Figures, Supplementary Notes and Supplementary References.
