# Supplementary material for: Enhanced oxidation resistance of active nanostructures via dynamic size effect
Source: Nat Commun. 2017 Feb 22;8:14459. doi: 10.1038/ncomms14459 (PMC5322499; doi:10.1038/ncomms14459)
Supplement: Supplementary Information — Supplementary Figures, Supplementary Notes and Supplementary References. [file ncomms14459-s1.pdf]

Supplementary Note 1. The interpretation of element-specific scanning tunneling microscopy (STM) images

Oxygen vacancy lines on the FeO surface could be used as a reference to elucidate the image contrast at the atomic scale. The same method for STM image interpretation has been used by Merte et al., who used atomic hydrogen to create oxygen vacancy loops on the FeO film supported on Pt(111)[1]. In our study, vacancy lines could be formed by the annealing of FeO islands in UHV at above 500 K. Comparing the STM image (Supplementary Fig. 3a) with the structural model (Supplementary Fig. 3b), we could identify unambiguously that top-layer oxygen atoms are resolved as protrusions in STM images taken at cryogenic temperatures. From the structural model, the formation of oxygen vacancy line causes oxygen atoms across the line being shifted by half a unit cell, and thus the separation of two oxygen domains, whereas Fe atoms remain unchanged in the same hexagonal lattice.

The modification of tip apex could cause the change in STM image contrast, resulting in element-specific STM images[2]. Merte et al. have shown explicitly that STM imaging of FeO on Pt(111) could exhibit a few element-specific imaging modes, resolving only Fe or O atoms or both as bright protrusions[2]. These image modes, could be easily selected in low temperature (LT) STM measurements by the controlled modification of tip apex. As described above, STM measurements with the bare W tip at cryogenic temperatures would result in the oxygen mode, i.e. oxygen atoms being resolved as protrusions. When a CO molecule adsorbed at the apex of W tip, the Fe mode, i.e. Fe atoms being resolved as protrusions, would be obtained at the sample bias between 10-100 mV. Supplementary Fig. 3c shows an Fe mode image, where bright protrusions are well aligned even across the vacancy line. The enhancement or reversal of chemical contrast induced by the adsorption of CO at the tip apex has often been reported in LT-STM studies [3,4].

The step structures of FeO islands could thus be viewed directly in the atomically resolved and element-specific STM images (Supplementary Fig. 3e-f). The combination of Fe-mode and O-mode STM images on the same FeO island reveals

both the number and stacking positions of Fe/O atoms and thus allow the construction of the structural model. Supplementary Fig. 3e and 3f show the atomic lattice shown in either imaging mode is ordered and strictly hexagonal, suggesting that a single element is resolved as protrusions. However, in contrast to 10 protrusions resolved at each edge of the island in the O mode image, 11 protrusions are clearly resolved at each edge in the Fe mode image. Note that, due to electron delocalization at elevated temperatures, STM imaging at above 200 K would display the mixed mode (Supplementary Fig. 3h), where both Fe and O atoms were resolved bright, or the Fe mode (Supplementary Fig. 3g) via the adsorption of CO at the tip apex. In the mixed mode STM images, both Fe and O atoms were imaged as bright protrusions but O atoms displayed a higher apparent height than Fe atoms.

Supplementary Note 2. The analysis of surface reconstruction in Fig. 3

In situ STM images presented in Fig. 3 shows that the surface reconstruction occurred after oxygen adsorbed at the edges. Before O_2 adsorption, 78 protrusions of Fe atoms in Fig. 3a and Supplementary Fig. 6a were imaged with 12 Fe atomic rows at each edge. After O_2 adsorption, the mixed mode STM image shows that the number of Fe atoms remain unchanged in O_2, but the number of O atoms has increased by 23 to 89 protrusions (Supplementary Fig. 6e). By comparing STM images before and after O_2 exposure, we found that the relative positions of Fe and O atoms have also changed. The change of relative positions of Fe and O atoms could also be evidenced by the line profiles across the same position of FeO surfaces, which clearly show the respective positions of Fe and O in the diagonal FeO unit cell (Supplementary Fig. 7a-f). O atoms display a higher apparent height than that of Fe atoms. Half of the three-fold hollow sites of the Fe lattice are filled with O, while the other half are empty and imaged as dark depressions. Upon O_2 exposure, O atoms have shifted, with respect to Fe atoms, to the adjacent three-fold hollow sites, as illustrated by the relative positions of Fe, O and empty hollow sites in the line profiles (Supplementary Fig. 7c, f). In addition, the line profile of the CUO step formed upon reconstruction

appears inverted to that of the CUF step (Supplementary Fig. 7g). Our results show the CUF-terminated $Fe_{78}O_{66}$ NS reconstructed completely to CUO-terminated $Fe_{78}O_{89}$ NS in O_2.

To understand the O-induced reconstruction of FeO NSs, DFT calculations were performed on an $Fe_{10}O_6$ cluster. Fig. 3h shows that the dissociation of O_2 at CUF sites leads to unstable oxygen adatoms, which tend to bind with neighboring CUF site to lower the edge energy. Feeling the stress from oxygen adatoms, oxygen atoms at their nearest neighbor would rotate around their neighboring Fe atoms by 60 degrees to the adjacent three-fold hollow sites of the Fe layer (only half of the three-fold hollow sites in the Fe layer are taken by O atoms in FeO). Such rotation propagates as the cascade movement of oxygen atoms across the surface plane. The motion of oxygen atoms follows a fixed transition path, which is usually observed in phase transformation. For small FeO islands, the cascade movement, initiated by oxygen from the step edges, is thorough, resulting all step edges being terminated by CUO atoms.

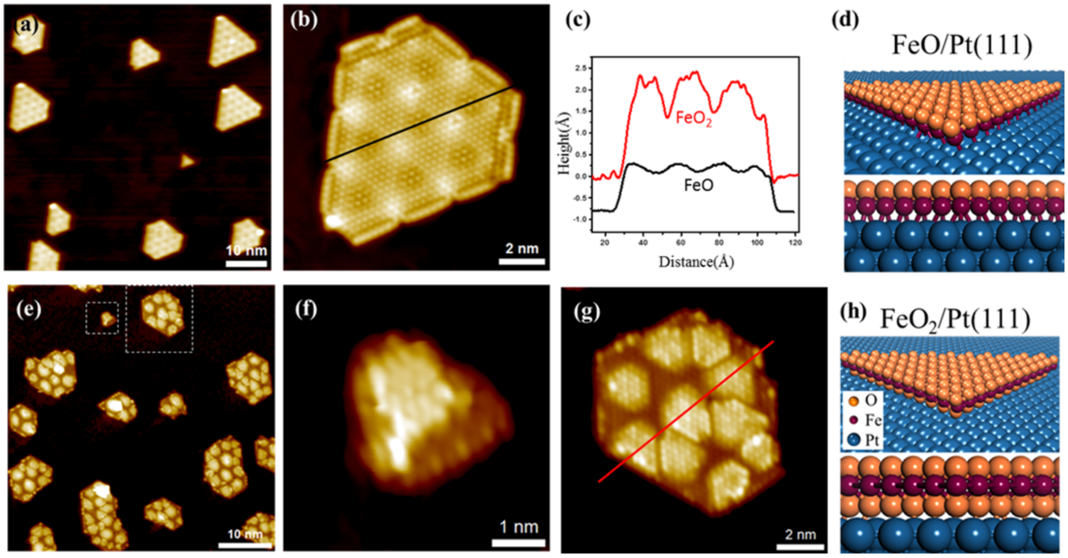

Supplementary Fig. 1 STM images of FeO NSs (a-b) and oxidized FeO NSs with FeO_2 domains (e-h). The as-prepared FeO/Pt(111) surface (a) was oxidized at 650-750 K in 2×10^{-5} mbar O_2 for 20 min to form the surface in (e).The line profiles marked in (b) and (g) are displayed in (c), which shows the clear differences in the apparent heights of FeO NS and FeO_2 domains. All FeO NSs in (e), regardless their sizes, were oxidized to form FeO_2 domains on the surface. Two typical oxidized FeO NSs, marked by the squares in (e), are magnified in (f) and (g), which have equivalent diameters of (f) 2.7 nm and (g) 8.2 nm, respectively. (d) and (h) display the structural models of FeO/Pt(111) and FeO_2/Pt(111), respectively. Bias voltage of sample (V_s) and tunneling currents (I_t): (b) V_s= +10 mV, I_t= 5 nA; (f) V_s= +200 mV, I_t= 0.38 nA; (g) V_s= +240 mV, I_t= 0.23 nA.

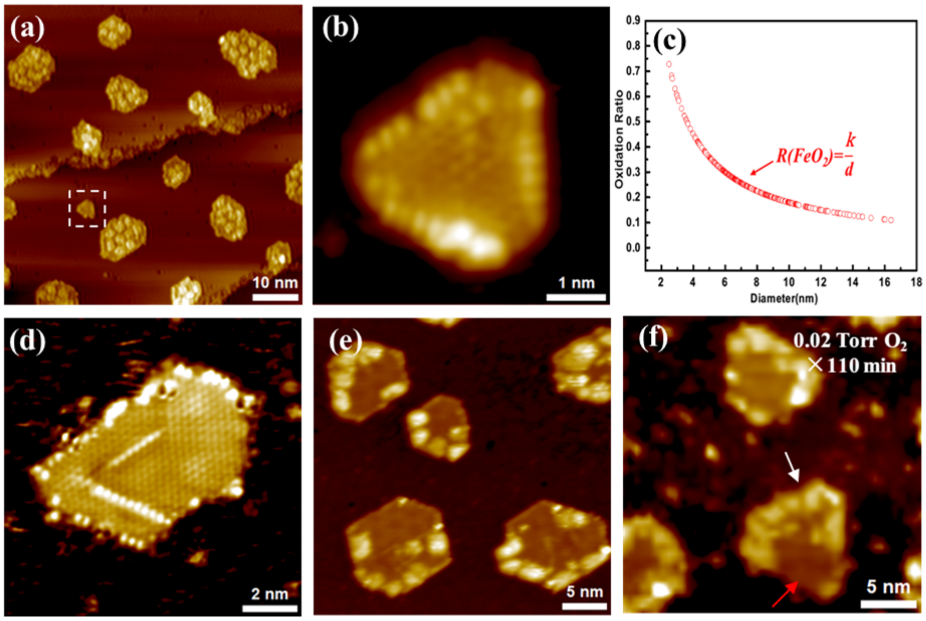

Supplementary Fig. 2 The oxidation of FeO NSs on Pt(111). (a) STM image of FeO NSs on Pt(111) after the annealing in 1×10^{-5} mbar O_2 at 500 K for 10 minutes. Most FeO NSs were oxidized to form FeO_2. An FeO island with an equivalent diameter of 3.1 nm remained at the FeO phase, which was marked by white square and magnified in (b). (c) Calculated size-dependent oxidation ratio by assuming a constant diffusion rate, *D*, for oxygen across the edge perimeter of FeO NSs and into the FeO/Pt interface. The development rate of FeO_2 domains could be written as: $\frac{dS(\mathrm{FeO_2})}{dt} = D\times\pi d$, where d is the equivalent diameter of the FeO island. The oxidation ratio could thus be derived as $R(FeO_2)=\frac{S(\mathrm{FeO_2})}{S}=\frac{k}{d}$, where k is the fitting parameter. Scanning parameters: (b) V_s= +67 mV, I_t= 2.6 nA; (d) V_s= +450 mV, I_t= 0.23 nA. (d-f) STM images of the developments of oxygen dislocation lines and FeO_2 domains along the step edges. (d) The exposure of 5×10^{-6} mbar O_2 at 300K led to only the formation of dislocation lines on the surface and the adsorption of oxygen at step edges. (e) STM image shows the anisotropic development of FeO_2 domains along the step edges during the oxidation in 5×10^{-6} mbar O_2 at 400 K. (f) In situ STM image obtained in 0.02 Torr O_2 at 300K shows the anisotropic development of FeO_2 domains along the step edges. (d-e) The oxidation of FeO NSs on Pt(111) after an annealing in 1×10^{-5} mbar O_2 at 500 K for 10 minutes.

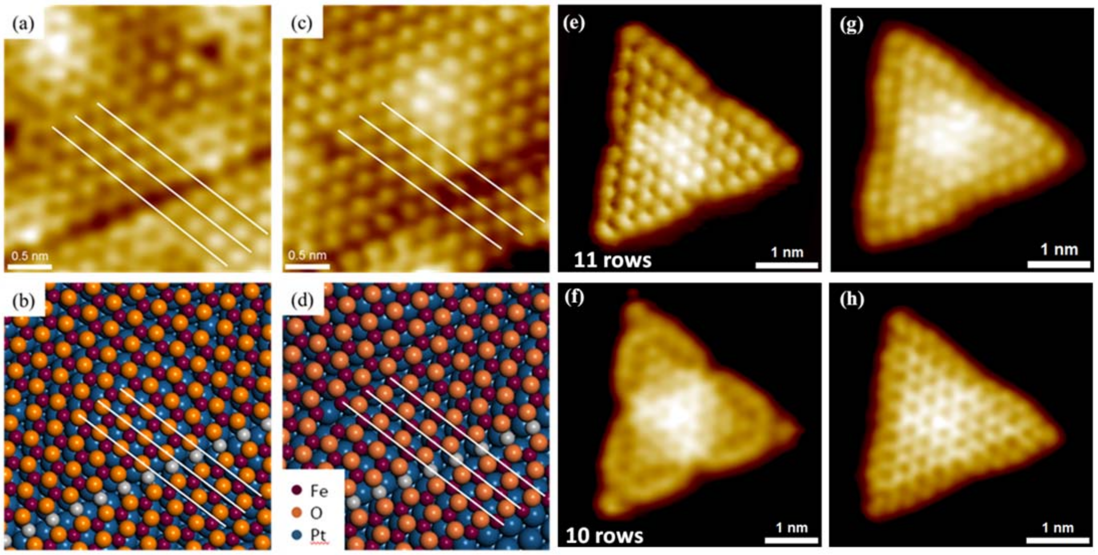

Supplementary Fig. 3 Interpretation of element-specific STM images. (a) O mode STM image, where oxygen atoms are resolved as protrusions. Across the oxygen vacancy line, the protrusions are shifted by half a unit cell. (b) The structural model of (a). (c) Fe mode STM image, where Fe atoms are resolved as protrusions. Across the oxygen vacancy line, the protrusions are still in line and Fe atoms at the oxygen vacancies could be resolved. (d) The structural model of (c). (a-f) are taken at 77 K. The Fe mode STM image was obtained by adsorbing a CO molecule at the tip apex. (e) and (f) show two typical element-specific STM images of $Fe_{66}O_{55}$ NS at 77 K which give the number and lattice of Fe or O atoms, respectively. (g) displays the typical Fe mode STM image of $Fe_{66}O_{55}$ NS at 270 K. The O mode STM image of $Fe_{66}O_{55}$ NS turned into a mixed mode image at 270 K (h) due to electron delocalization. Both Fe and O atoms were resolved, but O atoms display a higher apparent height than Fe atoms. Scanning parameters: (a) V_s= +18 mV, I_t= 1.2 nA; (c) V_s= +31 mV, I_t= 2.9 nA; (e) V_s= +7 mV, I_t= 4.5 nA; (f) V_s= +39 mV, I_t= 4.5 nA; (g) V_s= +20 mV, I_t=3.8 nA; (h)V_s= +32 mV, I_t=3.0 nA.

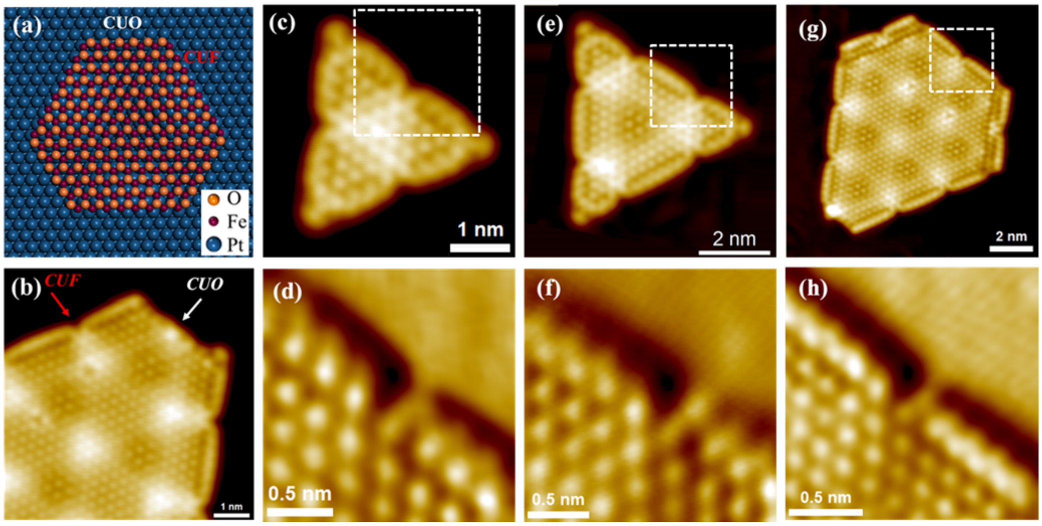

Supplementary Fig. 4 The edge structures of FeO NSs of different shapes and sizes on Pt(111). (a) The structural model of hexagonal FeO NS. Two step structures were observed for FeO NSs, which expose two-coordinated Fe or O atoms at the steps. These edge sites are often termed as coordinatively unsaturated ferrous (CUF) sites and coordinatively unsaturated oxygen (CUO) sites. The two types of steps are also noted as the CUF step and the CUO step, for simplification. (b) STM image of the CUF step and the CUO step. (c-h) The comparison of edge structures of FeO NPs of different sizes. The topographic (c, e and g) and the enlarged derivate (d, f and h) STM images show that the CUF edges are identical and independent of the islands size. The zigzag shape of step edge shows directly the coordinatively unsaturated ferrous (CUF) atoms in the outmost row. Scanning parameters: (b) V_s= +10 mV, I_t= 4.1 nA; (c) V_s= +7 mV, I_t= 4.5 nA; (e) V_s= +11 mV, I_t= 3.8 nA; (g) V_s= +10 mV, I_t= 5 nA.

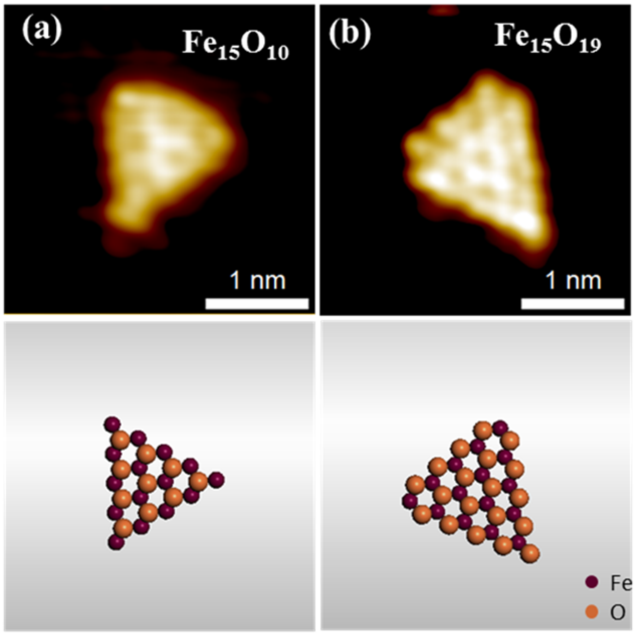

Supplementary Fig. 5 In situ STM images and the structural models of an $Fe_{15}O_{10}$ NS (a) before and (b) after the exposure of 1 × 10^{-9} mbar O_2 at 270 K. The $Fe_{15}O_{10}$ NS underwent a complete reconstruction to form the $Fe_{15}O_{19}$ NS. The energetically most favorable configuration of FeO NS on Pt(111), i.e. with both Fe and O atoms in the fcc positions of Pt(111), is dependent on the stacking sequences of Fe and O atoms with respect to Pt[5]. Monolayer FeO could display two Fe/O stacking sequences, as shown by the $Fe_{15}O_{10}$ and $Fe_{15}O_{19}$ NSs. Upon oxygen adsorption, the reconstruction of FeO NS causes O atoms to locate in the hcp positions of Pt(111). To reach the most stable configuration, CUO-terminated $Fe_{15}O_{19}$ NS need rotate by 60° to attain the optimized position on Pt(111), as shown in (b). Scanning parameters: (a) V_s= +130 mV, I_t= 0.58 nA; (b)V_s= +67 mV, I_t= 1 nA.

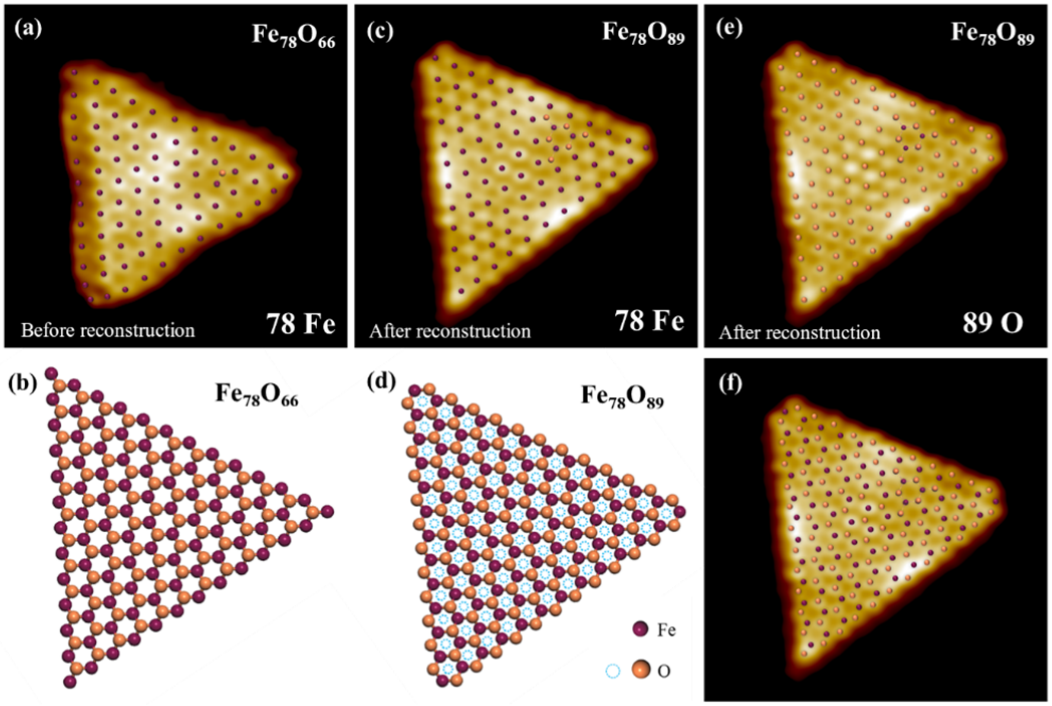

Supplementary Fig. 6 Image analysis of numbers of imaged protrusions of the $Fe_{78}O_{66}$ NS and $Fe_{78}O_{89}$ NS in Fig. 3. Before O_2 exposure, an Fe mode STM image of the $Fe_{78}O_{66}$ NS was shown in (a), with superimposed purple circles to mark the positions of Fe atoms. The corresponding structural model was shown in (b). After O_2 exposure, the mixed mode STM image of the $Fe_{78}O_{89}$ NS was shown in (c) and (e), with superimposed purple or blue circles to mark the positions of Fe or O atoms, respectively. The corresponding structural model was shown in (d). In (f), both purple and blue circles are displayed, showing the complete lattice of $Fe_{78}O_{89}$ NS, as in (d). The description on NS reconstruction is detailed in **Supplementary Note 2**. Scanning parameters: (a) V_s= +16 mV, I_t= 4.3 nA; (c,e,f) V_s= +80 mV, I_t=1.8 nA.

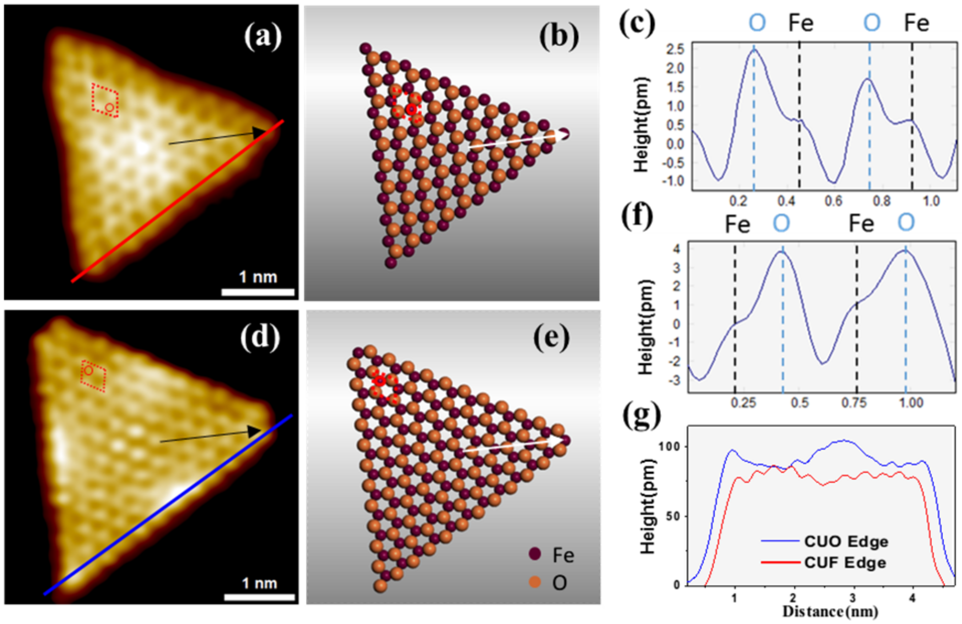

Supplementary Fig. 7 Image analysis of the change of relative positions of Fe and O atoms upon O_2 exposure. STM image of the FeO NS before O_2 exposure was displayed in (a), with the structural model depicted in (b). STM image of the FeO NS after O_2 exposure was displayed in (d), with the structural model depicted in (e). (c) and (f) plot the profiles of the arrow lines in (a) and (d), respectively. In the mixed mode STM images, O atoms display a higher apparent height than Fe atoms. The comparison of (c) and (f) clearly shows a shift in the relative positions between Fe and O atoms. The shift of O lattice could also be visualized by the position of Fe in the diagonal FeO unit cell, as illustrated by the red circles in (a) and (d). The comparison of line profiles along the step edges in (a) and (d) are plotted in (g). The line profile of the CUO-terminated step appears inverted to that of the CUF-terminated step. Scanning parameters: (a) V_s= +31 mV, I_t= 3.0 nA; (d) V_s= + 80 mV, I_t= 1.8 nA.

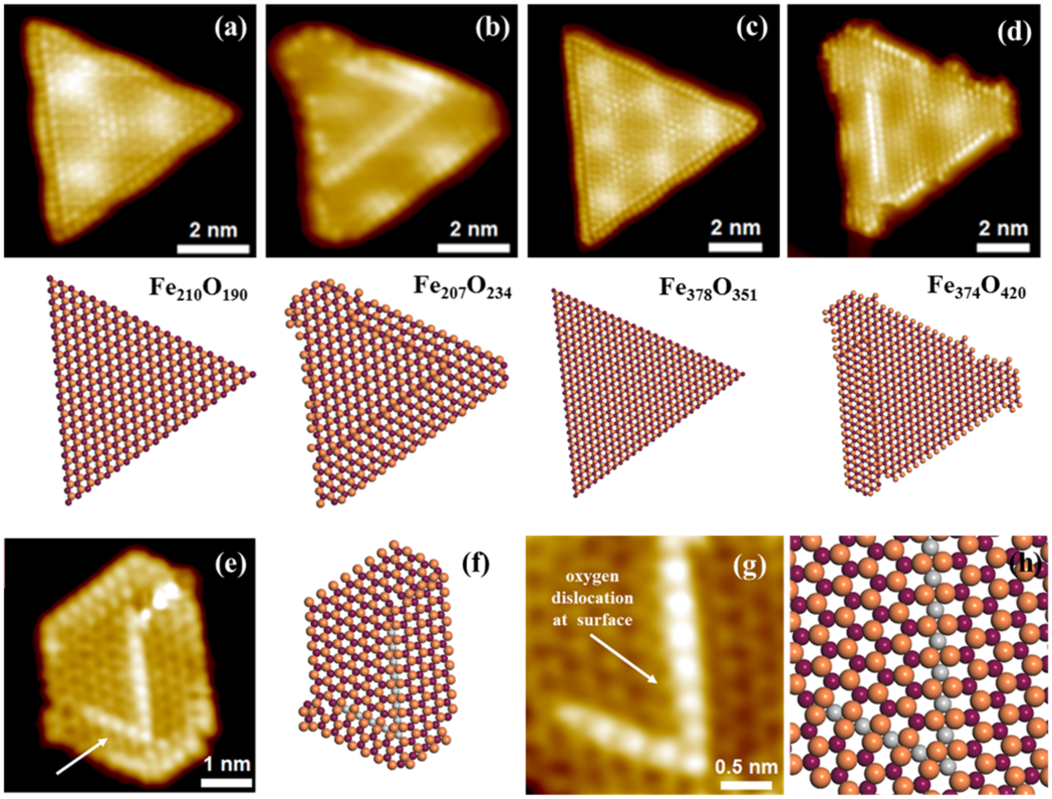

Supplementary Fig. 8 Partial reconstruction of FeO NSs with *d* > 3.2nm and the accompanying oxygen dislocations on FeO NSs. (a-d) In situ STM images of the $Fe_{210}O_{190}$ NS or $Fe_{378}O_{351}$ NS before (a, c) and after (b, d) the exposure of 1×10^{-9} mbar O_2 at 270 K. The $Fe_{210}O_{190}$ NS (a) in O_2 underwent a partial reconstruction and turn into an $Fe_{207}O_{234}$ island (b) with dislocation lines formed on the surface. The $Fe_{378}O_{351}$ NS underwent a partial reconstruction to form the $Fe_{374}O_{420}$ NS. The white protrusion lines on surface are oxygen dislocation lines, marking the boundary between the reconstructed domain and the unreconstructed domain. The color representations in the structural models are : Fe -purple and O -orange. (e-h) The atomic structure of oxygen dislocation lines on the FeO NS surface. (e) A mixed mode STM image of a hexagonal FeO NS. (f) The structure model of (e). The area with oxygen dislocation lines in (e) is magnified in (g), whose structure is depicted in (h). At the dislocation, Fe atoms become over-saturated with four-fold oxygen coordination and appear as protrusion lines running parallel to the steps in STM. Scanning parameters: (a) V_s= +20 mV, I_t= 3.4 nA; (b) V_s= +118 mV, I_t= 1.8 nA; (c) V_s= +18 mV, I_t= 3.7 nA; (d) V_s= +16 mV, I_t= 3.5 nA; (e) V_s= +210 mV, I_t= 0.8 nA; (g) V_s= +210 mV, I_t=0.8 nA.

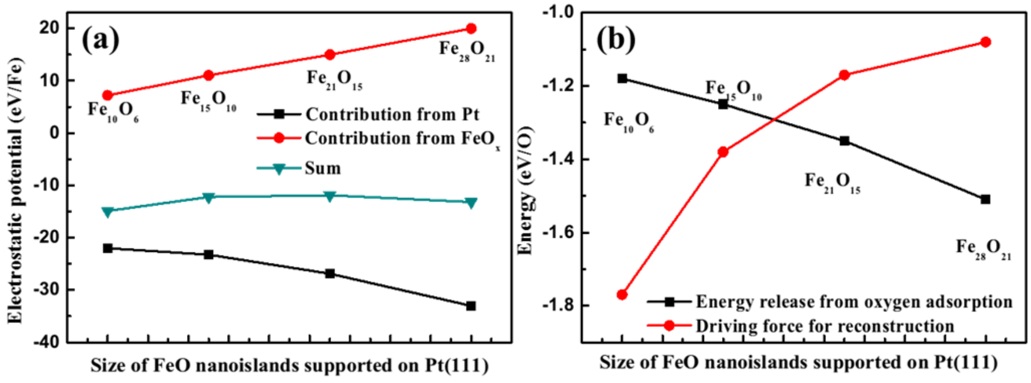

Supplementary Fig. 9 DFT calculated electronic properties and thermodynamics in the oxidation of FeO NSs. (a) DFT-calculated electrostatic potential at a CUF site of FeO NS supported on Pt(111). The electrostatic potential on a CUF site, which directly interacts with oxygen, is much lower for the small islands than the big ones if considering only the contribution from FeO. However, when supported on a metallic substrate, delocalized electron gas of the metal support provides sufficient metallic screening of the Coulomb repulsion within the FeO clusters, such that the size-dependence of the potential is greatly reduced/damped. (b) DFT-calculated energy associated with the oxidation and reconstruction of FeO NSs supported on Pt(111). Energy released from oxygen adsorption: the total energy released from oxygen adsorption divided by number of adsorbed oxygen atoms. The driving force for reconstruction: the total energy gained from oxygen-induced reconstruction divided by number of oxygen atoms on FeO islands.

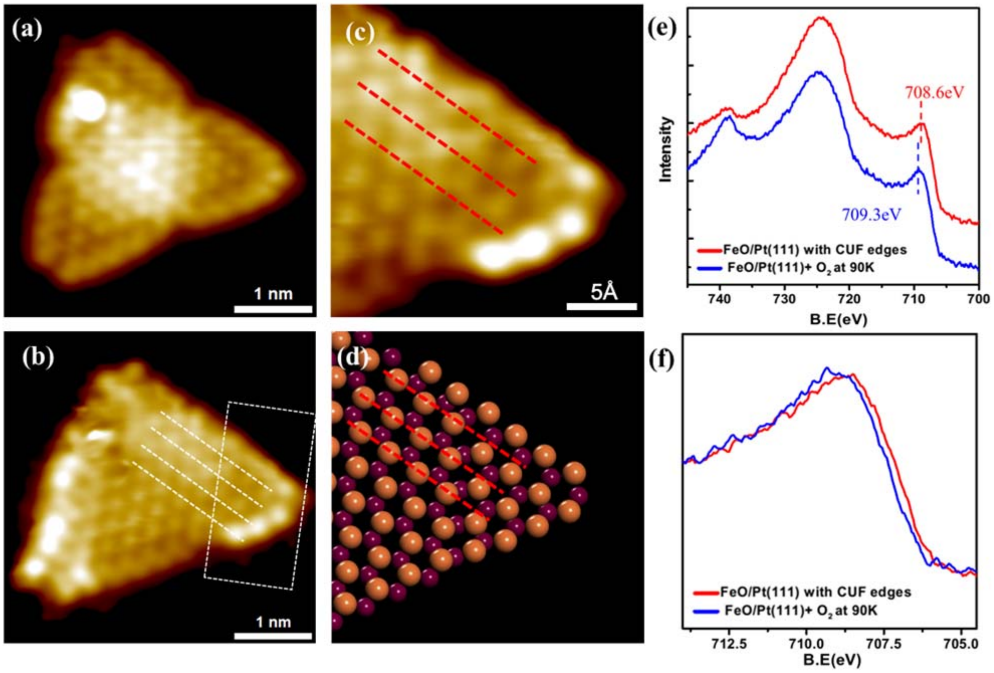

Supplementary Fig. 10 The interaction between FeO NS and O_2 at cryogenic temperatures. (a-b) In situ STM images of an FeO NS before (a) and after (b) the exposure of 1×10^{-9} mbar O_2 at 15 K. The white lines and the white square in (b) marked the region where reconstruction has taken place upon the dissociative adsorption of oxygen atoms at the edge. (c-d) The enlarged STM image and atomic structure model of the reconstructed region of FeO surface. Scanning parameters: (a) V_s= +7 mV, I_t= 3.1 nA; (b, c) V_s= +7 mV, I_t= 6.2 nA. (e) Fe 2p XPS spectra of FeO/Pt(111) before and after the O_2 exposure at 90 K. The binding energy of Fe $2p_{3/2}$ shifts from 708.6 eV to 709.3 eV upon O_2 exposure. (f) Magnified XPS spectra of Fe $2p_{3/2}$ before (red) and after (blue) O_2 exposure. XPS spectra have subtracted the spectrum of Pt(111) to minimize the influence of secondary electron background.

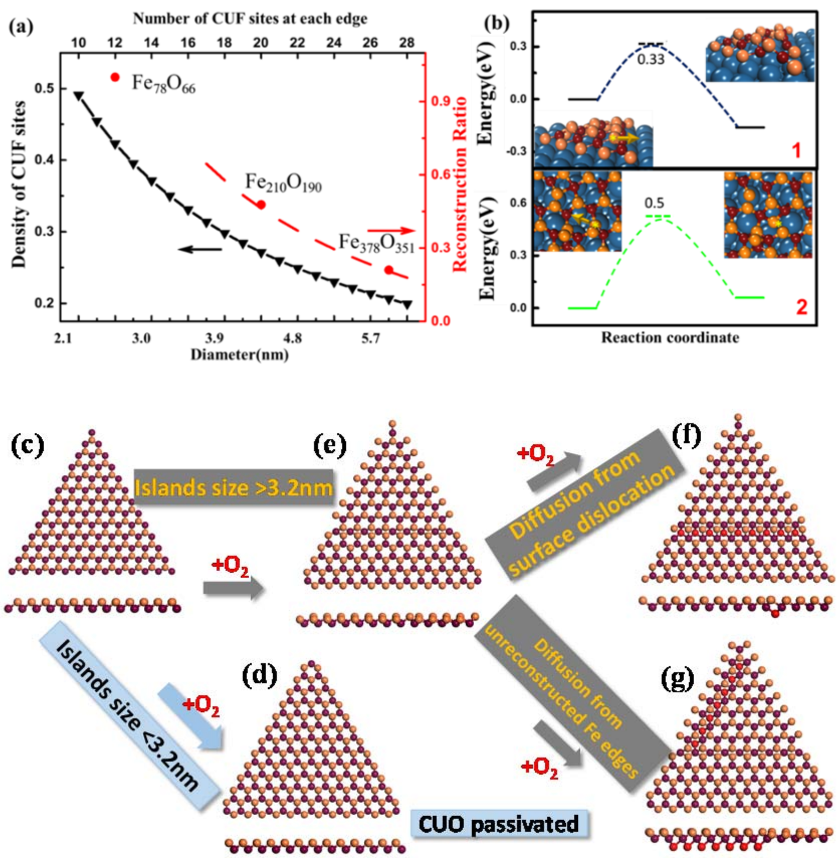

Supplementary Fig. 11 The size-dependent reconstruction of FeO NSs. (a) The size-dependent reconstruction of FeO NSs (red) and its relation with the density of CUF sites (black). The reconstruction ratio (*R(d)*) measured for $Fe_{78}O_{66}$, $Fe_{210}O_{190}$, and $Fe_{378}O_{351}$ NSs. *R(d)* is defined as the number ratio of shifted oxygen atoms over total surface oxygen atoms. Here, the density of CUF sites is defined as the number ratio of CUF sites over total Fe sites in the NS and triangular FeO NSs were used as the model structure. (b) Potential energy diagrams depicting the mode of action for oxygen diffusion. The diffusion pathways and barriers are displayed for oxygen atom moving to the adjacent hollow site of the Fe layer (**1**) and for oxygen penetrating into the FeO-Pt(111) interface from surface dislocation (**2**). (c-g) The schematic illustration of dynamic size effect and its influence on the oxidation kinetics of FeO NSs. (c-d) The complete reconstruction of FeO NS with $d < 3.2$ nm, which passivates the pathway of edge oxygen penetrating into the interface. FeO NSs with $d < 3.2$ nm thus exhibit enhanced resistance to oxidation. (e-g) The situation for FeO NS with $d >$ 3.2 nm, which underwent a partial reconstruction (e), accompanied by the development of oxygen dislocation lines on the surface. Subsequently, oxygen atoms could diffuse from the dislocation line (f) or from the unreconstructed step edge (g), leading to the onset formation of FeO_2 domains.

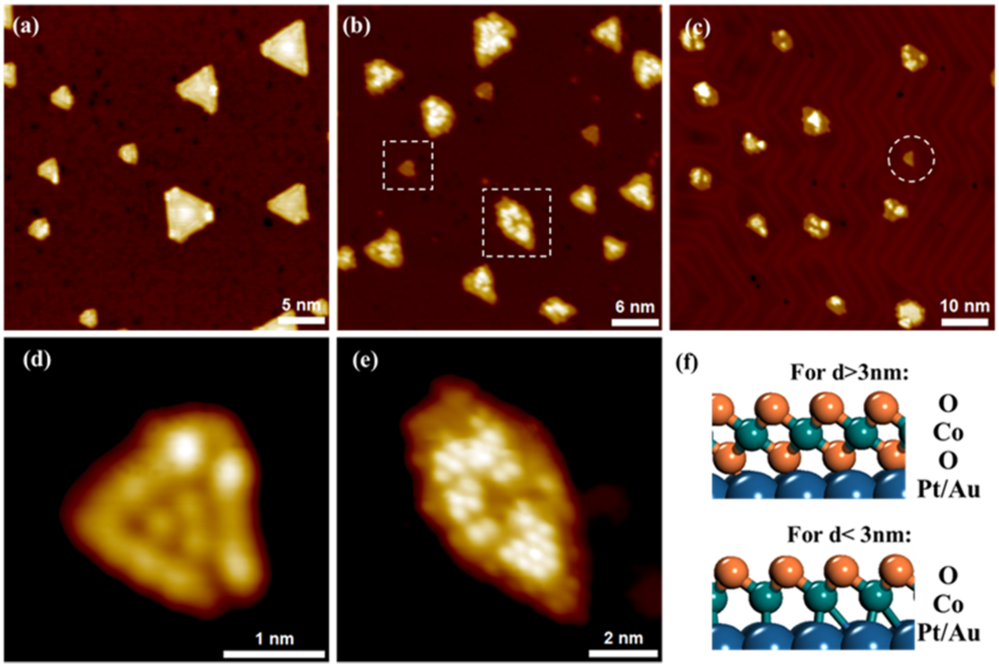

Supplementary Fig. 12 The size-dependent oxidation kinetics of CoO NSs on Pt(111) and Au(111). (a-b) STM images of the the CoO/Pt(111) surface after the annealing in 1×10^{-5} mbar O_2 at 500 K for 10 minutes. Most CoO NSs were oxidized to form CoO_2 domains, except for CoO NSs with $d < 3$ nm. (c) STM image of the CoO/Au(111) surface after annealing in 1×10^{-7} mbar O_2 at 600 K for 10 minutes. (d-e) Atomic STM images of an CoO NS ($d = 2.0$ nm) on Pt(111), which remained the CoO phase, and of an oxidized CoO NS ($d = 5.6$ nm) with the formation of CoO_2 domains. (f) Models illustrating the size-dependent oxidation of CoO NSs on Pt(111)/Au(111). Scanning parameters: (d) V_s= +53 mV, I_t= 2.1 nA; (e) V_s= +300 mV, I_t= 0.12 nA.

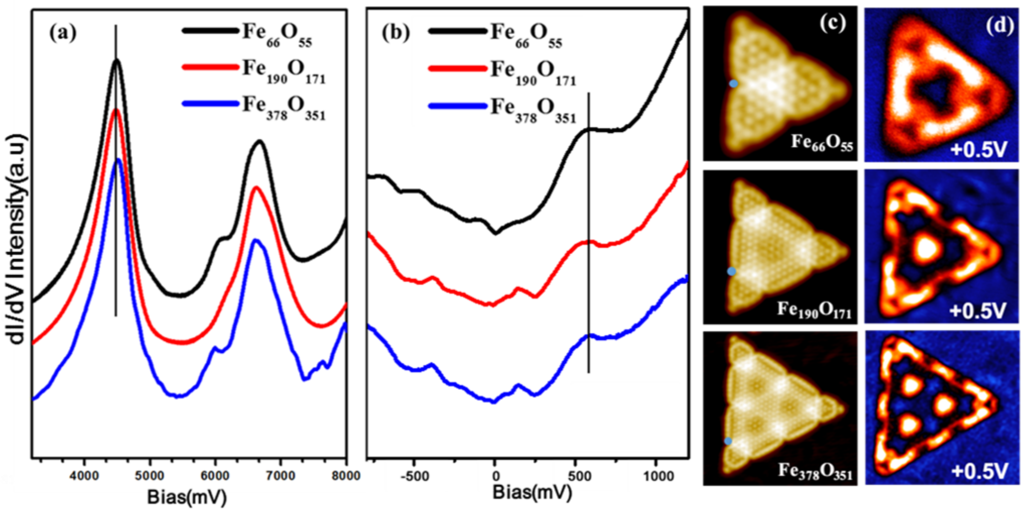

Supplementary Fig. 13 Electronic properties of FeO NSs supported on Pt(111). (a-b) Differential conductance (*dI/dV*) spectra taken in the field emission regime (a) or near the Fermi level (b) at the CUF sites of $Fe_{66}O_{55}$, $Fe_{190}O_{171}$ and $Fe_{378}O_{351}$. The positions are marked by the blue dots in the corresponding STM images in (c). The corresponding differential conductance (*dI/dV*) maps of $Fe_{66}O_{55}$, $Fe_{190}O_{171}$ and $Fe_{378}O_{351}$ are shown in (d). Modulation frequency = 413 Hz, amplitude = 20 mV (peak to peak).

Note: Local work function (LWF) could be derived from the position of the resonance peak at the lowest energy in the close-loop d*I*/d*V* spectra, taken in the field emission region (FER)[6,7]. It is clearly shown that LWFs at the same position are independent of NS size, when the equivalent diameter of FeO NS is larger than 2 nm. The *dI/dV* spectra taken near the Fermi level provide a comparison for local density of states (LDOS). The LDOS peaks at ~560 meV above the Fermi level correspond to the electronic states contributed by the Fe dz^2 orbitals. The electronic properties of CUF sites, as indicated by LWF and LDOS measurements, were found the same for FeO islands with $d > 2$ nm.

Supplementary References

1. Merte, L. R. *et al.* Correlating STM contrast and atomic-scale structure by chemical modification: Vacancy dislocation loops on FeO/Pt(111). *Surf Sci* **603**, L15-L18, http://dx.doi.org/10.1016/j.susc.2008.11.014 (2009).
2. Merte, L. R. *et al.* Tip-Dependent Scanning Tunneling Microscopy Imaging of Ultrathin FeO Films on Pt(111). *J. Phys. Chem. C* **115**, 2089-2099, http://dx.doi.org/10.1021/jp109581a (2011).
3. Bartels, L., Meyer, G. & Rieder, K.-H. Controlled vertical manipulation of single CO molecules with the scanning tunneling microscope: A route to chemical contrast. *Appl Phys Lett* **71**, 213-215, http://dx.doi.org/10.1063/1.119503 (1997).
4. Hahn, J. & Ho, W. Single Molecule Imaging and Vibrational Spectroscopy with a Chemically Modified Tip of a Scanning Tunneling Microscope. *Phys Rev Lett* **87**, 196102, http://dx.doi.org/10.1103/PhysRevLett.87.196102 (2001).
5. Kim, Y. J. *et al.* Interlayer interactions in epitaxial oxide growth: FeO on Pt(111). *Phys. Rev. B* **55**, R13448-R13451, http://dx.doi.org/10.1103/PhysRevB.55.R13448 (1997).
6. Dougherty, D. B. *et al.* Tunneling spectroscopy of Stark-shifted image potential states on Cu and Au surfaces. *Phys Rev B* **76**, 125428, http://dx.doi.org/10.1103/PhysRevB.76.125428 (2007).
7. Rienks, E. D. L., Nilius, N., Rust, H.-P. & Freund, H.-J. Surface potential of a polar oxide film: FeO on Pt(111). *Phys Rev B* **71**, 241404, http://dx.doi.org/10.1103/PhysRevB.71.241404 (2005).
